# Development of a rapid on-site nucleic acid detection method for new genotype muscovy duck parvovirus based on RPA-CRISPR/Cas12a

**DOI:** 10.3389/fvets.2025.1621697

**Published:** 2025-08-26

**Authors:** Qizhang Liang, Wei Chen, Weiwei Wang, Rongchang Liu, Qiuling Fu, Guanghua Fu, Longfei Cheng, Nansong Jiang, Hongmei Chen, Yu Huang

**Affiliations:** Institute of Animal Husbandry and Veterinary Medicine, Fujian Academy of Agricultural Sciences, Fuzhou, China

**Keywords:** N-MDPV, RPA, LFS, CRISPR/Cas12a, on-site detection

## Abstract

New genotype Muscovy Duck Parvovirus (N-MDPV), a member of the Parvoviridae family, exhibits broad host tropism affecting Muscovy ducks, semi-Muscovy ducks, and white Kaiva duck. This pathogen causes severe morbidity and mortality in ducklings under 3 weeks of age, characterized by classic parvoviral lesions, beak atrophy, and growth retardation, posing substantial economic threats to China’s duck industry. To address diagnostic challenges, we developed an equipment-free detection platform targeting the conserved VP3 gene of N-MDPV. By integrating recombinase polymerase amplification (RPA) with CRISPR/Cas12a-mediated lateral flow strip (LFS) visualization, this method achieved isothermal amplification at 37°C within 35 min, eliminating dependency on thermocyclers. Validation experiments demonstrated exceptional sensitivity with a detection limit of 1.3 gene copies. Specificity testing revealed no cross-reactivity with eight common avian pathogens, confirming target exclusivity. Clinical validation using 98 field-collected duck tissue samples showed 98.98% concordance between our RPA-CRISPR/Cas12a-LFS and quantitative PCR. This study establishes the first CRISPR/Cas12a-based on-site diagnostic tool for N-MDPV, combining rapidity, sensitivity, accuracy and field-deployability.

## 1 Introduction

Muscovy duck parvovirus (MDPV), the causative agent of Muscovy duck parvovirus disease (commonly termed “three-week disease”), is a highly contagious pathogen exclusively infecting *Cairina moschata* (Muscovy ducks) (1, 2). It leads to open-mouthed breathing and diarrhea in infected ducks, and in fatal cases, pulmonary hemorrhage, pancreatic hemorrhage, and/or white necrotic spots, as well as duodenal mucosal bleeding. It primarily results in the illness and death of Muscovy ducks within 3 weeks of age (3).

In 2008, an emergent syndrome characterized by beak atrophy and growth retardation was reported in semi-Muscovy ducks in Fujian Province, China (4). In May 2012, 19-day-old Muscovy ducklings on a Shanghai suburban farm exhibited mass symptoms including watery diarrhea, wheezing and locomotor dysfunction. Though clinical signs, gross lesions and disease course resembled those of previously described MDPV infections (5), morbidity and mortality rates were significantly higher. Genome sequencing of the isolated strain SAAS-SHNH revealed 93.7% nucleotide identity with MDPV strain FM (NC_006147), along with two putative recombination events in the 419–610 nt and 3,113–4,241 nt regions—providing the first evidence of recombination between MDPVs and GPVs (6). Epidemiological and pathogen studies identified this as a new genotype, named New-genotype Muscovy Duck Parvovirus (N-MDPV), based on its significant differences from MDPV in genome, host range, antigenicity, and pathogenicity (4, 7). It should be specifically noted that, due to its recombinant properties, this virus is also frequently referred to as “recombinant Muscovy duck parvovirus (3, 8, 9).

N-MDPV demonstrates expanded host specificity, infecting not only Muscovy ducks but also semi-Muscovy ducks and white Kaiva duck; In terms of pathogenicity, it not only induces lesions typical of the classical Muscovy duck parvovirus, but also causes short beaks and growth retardation in infected ducks (4, 6–8), posing significant harm and economic losses to China’s duck industry. Therefore, the detection method established in this study holds great significance.

In early disease identification and diagnosis, molecular diagnostic approaches targeting nucleic acids demonstrate superior efficacy over conventional methods that detect pathogen-derived antibodies or antigens (10, 11). CRISPR/Cas12a-based detection platforms have gained significant traction in clinical diagnostics due to their exceptional sequence specificity for pathogen identification (12). This system operates through crRNA-guided recognition of double-stranded DNA sequences containing a protospacer adjacent motif (PAM), which triggers Cas12a activation. The activated enzyme subsequently cleaves fluorophore-quencher complexes in reporter substrates, producing measurable fluorescent signals (13). The synergistic integration of recombinase polymerase amplification (RPA) with CRISPR/Cas12a technology (RPA-CRISPR/Cas12a) enhances diagnostic precision by mitigating false-positive results inherent to standalone RPA while amplifying the CRISPR-mediated cleavage signal (14, 15). This combined methodology has been successfully adapted for lateral flow strip (LFS) platforms, enabling rapid, sensitive, and specific on-site visual detection (16, 17).

Duck parvoviruses have a linear single-stranded DNA genome about 5.1 kilobases long, with two main open reading frames (ORFs) and inverted terminal repeats (ITRs) at each end. The left ORF encodes non-structural proteins (NS1/NS2) for viral replication, while the right ORF produces three overlapping structural proteins (VP1, VP2, VP3) via differential splicing (18, 19). The VP3 gene demonstrates high conservation across waterfowl parvoviruses, particularly in its structural epitope regions, making it an optimal target for nucleic acid-based detection methods (9, 19, 20). This sequence stability ensures reliable diagnostic performance across viral variants.

In this study, we evaluated the effectiveness of different combinations of RPA primers, probes, and crRNA targeting the N-MDPV VP3 gene. Subsequently, we assessed the sensitivity and specificity of the optimized RPA-CRISPR/Cas12a-LFS method. The method was also employed to analyze clinical samples, revealing 98.98% concordance with results obtained through conventional quantitative polymerase chain reaction (qPCR). This equipment-free detection platform, requiring only a heating block and lateral flow strips, provides a field-deployable solution for rapid on-site diagnosis and epidemiological monitoring of N-MDPV infections.

## 2 Materials and methods

### 2.1 Clinical samples, reagents, plasmid and instruments

98 samples of Muscovy ducks (*Cairina moschata*) suspected of N-MDPV infection were collected in Fujian Province. RNA and DNA extraction was performed using the Animal Total RNA/DNA Isolation Kit from TianLong (Suzhou, China). The LbCas12a protein (a member of the Cas family of proteins and comes from Lachnospiraceae bacteria), EcoRI, and XbaI endonucleases were purchased from New England Biolabs (MA, United States). The RPA kit and LFS was obtained from EZassay Ltd. (Shenzhen, China). The VP3 gene was amplified from N-MDPV genome DNA (strain FJM3, GenBank No. KR075690.1) and inserted into pcDNA3.1 with a Flag tag. A constant temperature metal bath, purchased from Gingko Biotech (Beijing, China), was set at 37°C for the experiments. Gel imaging was carried out using equipment from Gene Company Limited (MA, United States). DNA and RNA concentrations were measured with the Nanodrop ND-2000 spectrophotometer (NanoDrop Technologies, DE), and fluorescence intensity was measured using the Tecan Infinite M200 plate reader (Männedorf, Sweden).

### 2.2 Design and screening for primers and crRNA of RPA

We utilized the web-based RPA Design platform^1^ to generate four primer pairs specific to the VP3 gene of N-MDPV (FJM3 strain, GenBank KR075690.1), with amplicon sizes constrained between 150 and 250 bp and primer lengths configured at 25–35 nucleotides. For Cas12a targeting, crRNA constructs were engineered to recognize sequences immediately downstream of 5’-TTTV-3′ protospacer adjacent motifs (PAMs). Each crRNA comprised: a 5′-T7 promoter sequence (UAAUACGACUCACUAUA), a Cas12a-binding scaffold (UAAUUUCUACUAAGUGUAGAU), and a variable 20–25 nt target sequence following the PAM motif. The crRNA Design interface^2^ facilitated sequence optimization through predictive scoring algorithms. Synthesis of all oligonucleotides was commercially outsourced to EZassay Ltd. (Shenzhen, China). Complete nucleic acid sequences have been archived in Table 1.

**Table 1:** The sequence of RPA primers and crRNA.

Name	Sequence (5′-3′)	Position
N-MDPV-VP3-RPA-F1	ACTCACACAGAAGCAGAGGCTTCCAGCATCC	1,001–1,031
N-MDPV-VP3-RPA-R1	GGAGCTCTAGTAGTGTTTTGTTCATTCGTTA	1,173–1,203
N-MDPV-VP3-RPA-R2	CTGAACTCGTAGGAGCTCTAGTAGTGTTTTG	1,184–1,214
N-MDPV-VP3-RPA-R3	ATCAAGATCTGAACTCGTAGGAGCTCTAGTA	1,192–1,222
N-MDPV-VP3-crRNA	UAAUUUCUACUAAGUGUAGAUGCUAAAGAUCCAUACAGAUCUG	1,055–1,076

### 2.3 RPA reactions

RPA amplification was conducted using manufacturer-recommended parameters with the commercial RPA Kit. Each 20 μl reaction mixture contained 10.0 μl reaction buffer, 6.0 μl nuclease-free ddH_2_O, and 0.5 μl each of 20 μM forward/reverse primers. The assembled reactions underwent thermal incubation at 37°C for 20 min in a metal bath.

### 2.4 Cas12a detection reactions

The CRISPR/Cas12a detection system comprised 5 μl RPA products, 1 μl LbCas12a protein, 2 μl Cas12a reaction buffer, 1 μl crRNA, and 0.6 μl fluorophore-quencher modified ssDNA reporter (FAM-TTATT-BHQ, 4 μM) in a 20 μl reaction volume. After thermal treatment at 37°C for 15 min in a metal bath, enzymatic activity was quantified through fluorescence detection using excitation/emission wavelengths of 492/521 nm. The dual-functional probe system (FAM-TTATT-Biotin and FAM-TTATT-BHQ) was synthesized via HPLC purification by EZassay Ltd. (Shenzhen, China).

### 2.5 Lateral flow detection

For lateral flow strip (LFS) analysis, 2 μl reaction product was combined with 78 μl dilution buffer and assessed following a 2-min incubation with LFS at ambient temperature. Results were interpreted via clearance-based detection principle: a solitary band at the control line (C) indicated positivity, whereas concurrent C and test line (T) bands confirmed negativity.

### 2.6 Optimization of CRISPR/Cas12a reaction parameters

To optimize the working concentrations of Cas12a and crRNA, parameter combinationsof Cas12a (25, 50, 100, 150 and 200 nmol/L) and crRNA (50, 100, 150 and 200 nmol/L) were systematically tested, with fluorescence quantification establishing 200 nmol/L and 50 nmol/L as optimal for Cas12a and crRNA, respectively.

### 2.7 Analytical sensitivity and diagnostic specificity of the RPA-CRISPR/Cas12a assay

To investigate the sensitivity of the RPA-CRISPR/Cas12a reaction, 10-fold serial dilutions of the pcDNA3.1-N-MDPV VP3-Flag plasmid standard were used as templates for the RPA reaction, whereas ddH2O was used as a negative control. The nucleic acids extracted from multiple viruses that seriously threaten poultry health, namely, Duck adenovirus 3 (DAdV-3), Fowl adenovirus type 4 (FAdV-4), Duck astrovirus (DAstV), Duck plague virus (DPV), Duck hepatitis virus (DHV), Duck circovirus (DuCV), Duck tembusu virus (DTMUV) and Duck reovirus (DRV) were detected to evaluate the analytical specificity of the RPA-CRISPR/Cas12a assay.

### 2.8 Statistical analysis

GraphPad Prism 9 software (GraphPad Software, Inc.) was utilized to analyze the data. Statistical significance was evaluated through the application of two-tailed t-tests. The results were shown as mean ± standard error of the mean (SEM) based on three independent experiments, with *p* values lower than 0.05 being considered significant.

## 3 Results

### 3.1 Mechanism of the elimination method for strip detection

**Figure 1:**
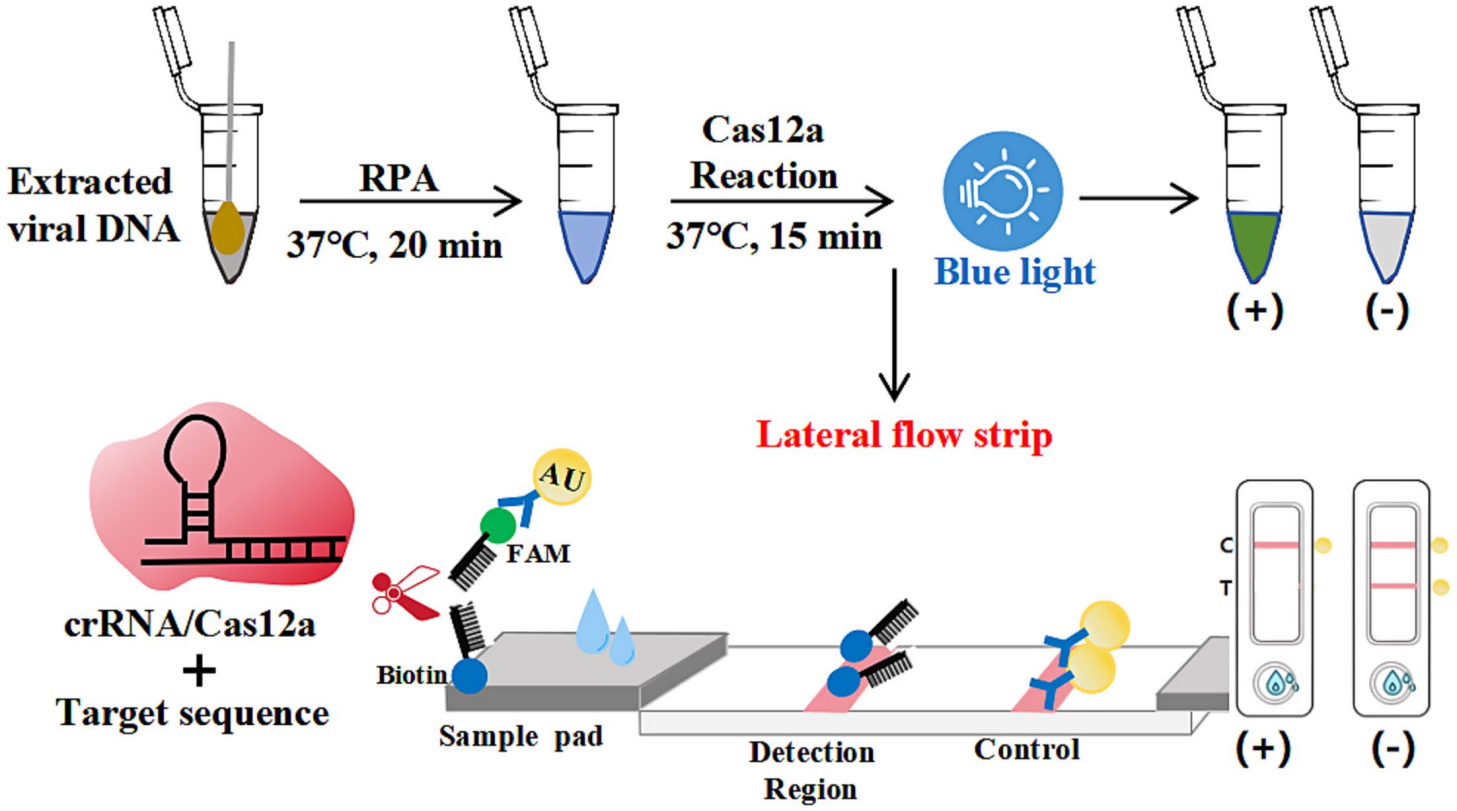
Schematic diagram of the elimination method for strip detection. The dual-labeled ssDNA probe (5’-FAM-Ab-gold/3′-biotin) enabled lateral flow strip (LFS) analysis, with upper segments depicting inactive conformations and lower segments indicating activated states.

As schematized in Figure 1, colloidal gold-FAM antibody conjugates formed a signaling complex with dual-functional probes (5’-FAM-ssDNA-Biotin-3′). Immobilized streptavidin on the control line (C) captured the biotinylated detection complex, generating a visible red band. In positive samples containing N-MDPV DNA, Cas12a’s collateral cleavage activity severed the ssDNA reporter, preventing colloidal gold-FAM conjugates from reaching the test line (T)—manifested as C-line band only. Negative samples preserved intact probes, producing both C and T-line bands. Absence of C-line bands indicated invalid tests due to insufficient sample migration or reagent failure. This cleavage-mediated signal elimination mechanism achieved visual interpretation without instrumentation.

### 3.2 RPA primer screening

**Figure 2:**
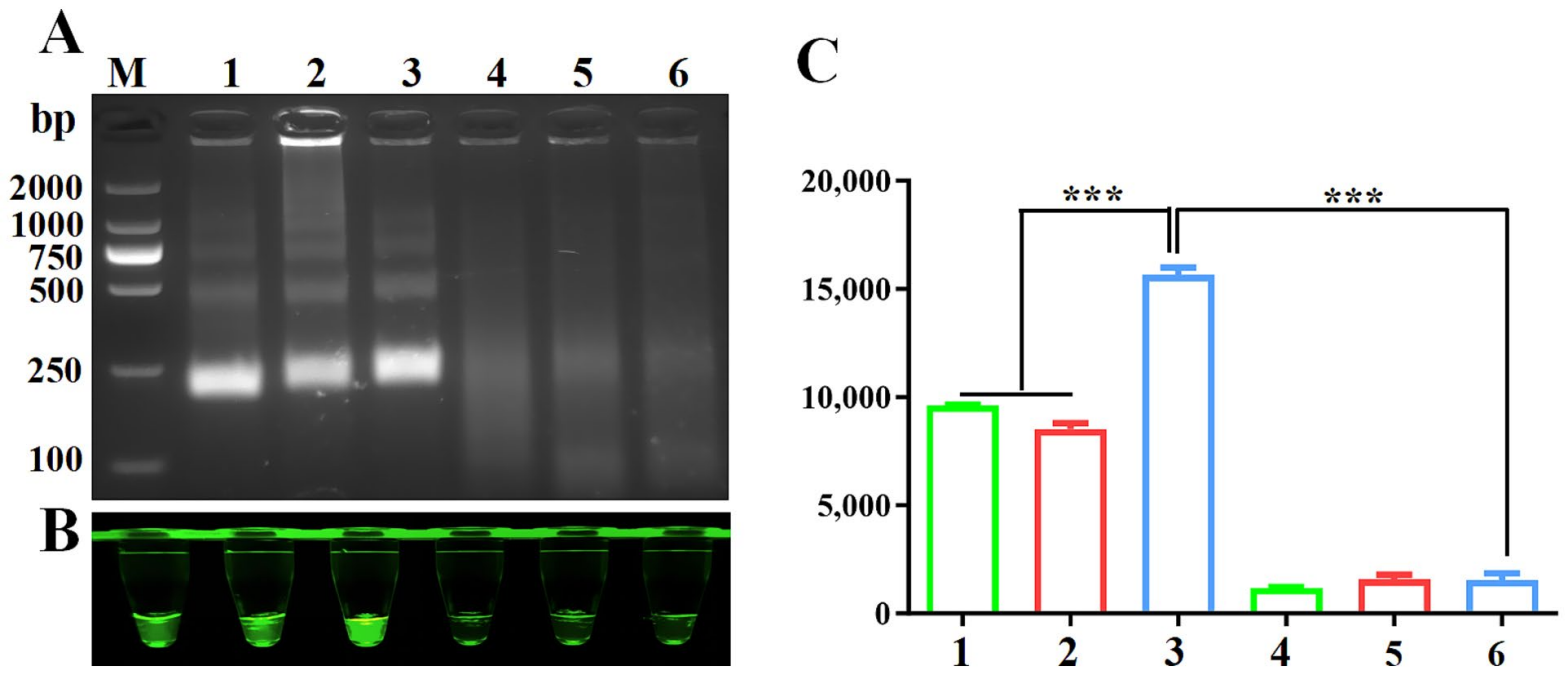
Screening of RPA primers. **(A)** RPA products verified by 1% agarose gel electrophoresis. M: DL2000 DNA marker; 1: F1R1; 2: F1R2; 3: F1R3; 4: F1R1-H_2_0; 5: F1R2-H_2_0; 6: F1R3-H_2_0;. Negative control using water as template was included in each reaction. CRISPR/Cas12a fluorescence detection were performed using three primers set. Fluorescence intensity **(B)** and fluorescence values **(C)** were shown for CRISPR/Cas12a detection using three primer sets.

Proper design of primer sequences plays a critical role in RPA performance. Initial screening of three VP3-targeting primer pairs revealed successful amplification, with agarose gel electrophoresis confirming expected 250 bp amplicons across all primer groups (Figure 2A). Cas12a-LFS analysis demonstrated positive signals for all primer pairs (Figure 2B, lanes 1/2/3) with negative control remaining blank. Fluorescence quantification (Figures 2B lower panel, 2C) identified primer set #3 as the top performer through maximum signal intensity and values. This optimized primer pair was consequently chosen for subsequent assay development.

### 3.3 Optimization of Cas12a and crRNA concentrations

**Figure 3:**
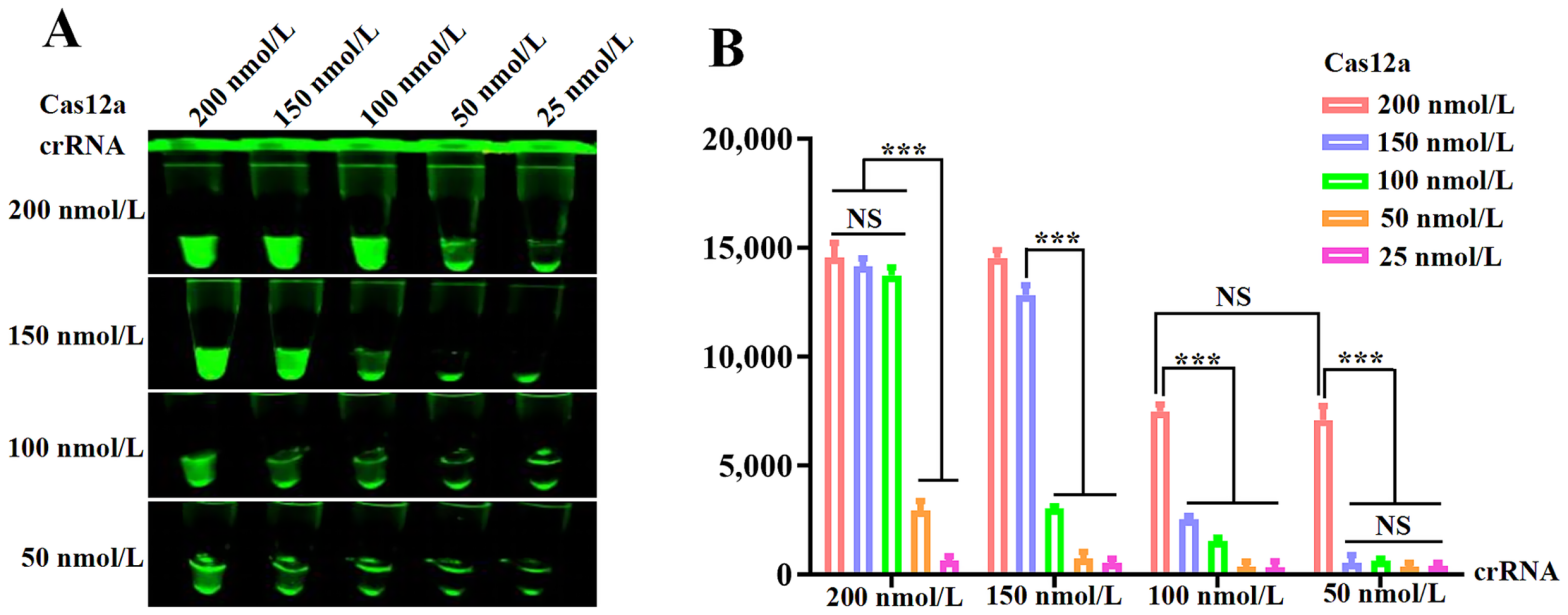
Optimization of the RPA and CRISPR/Cas12a system. **(A)** Fluorescence intensity based on CRISPR/Cas12a reaction mediated by different concentrations of Cas12a and crRNA. **(B)** Measurement of fluorescence values using a fluorescence microplate reader.

To determine optimal Cas12a and crRNA concentrations, gradient combinations of Cas12a (25–200 nmol/L) and crRNA (50–200 nmol/L) were systematically evaluated. Figure 3A demonstrates that 200 nmol/L Cas12a generated stronger fluorescence signals than 150 nmol/L, while 100 nmol/L crRNA did not outperform 50 nmol/L under identical conditions. Fluorescence quantification (Figure 3B) indicated no statistically significant differences across higher concentration groups. Consequently, 200 nmol/L Cas12a and 50 nmol/L crRNA were selected as optimal parameters for downstream assays.

### 3.4 Sensitivity of RPA-CRISPR/Cas12a method

**Figure 4:**
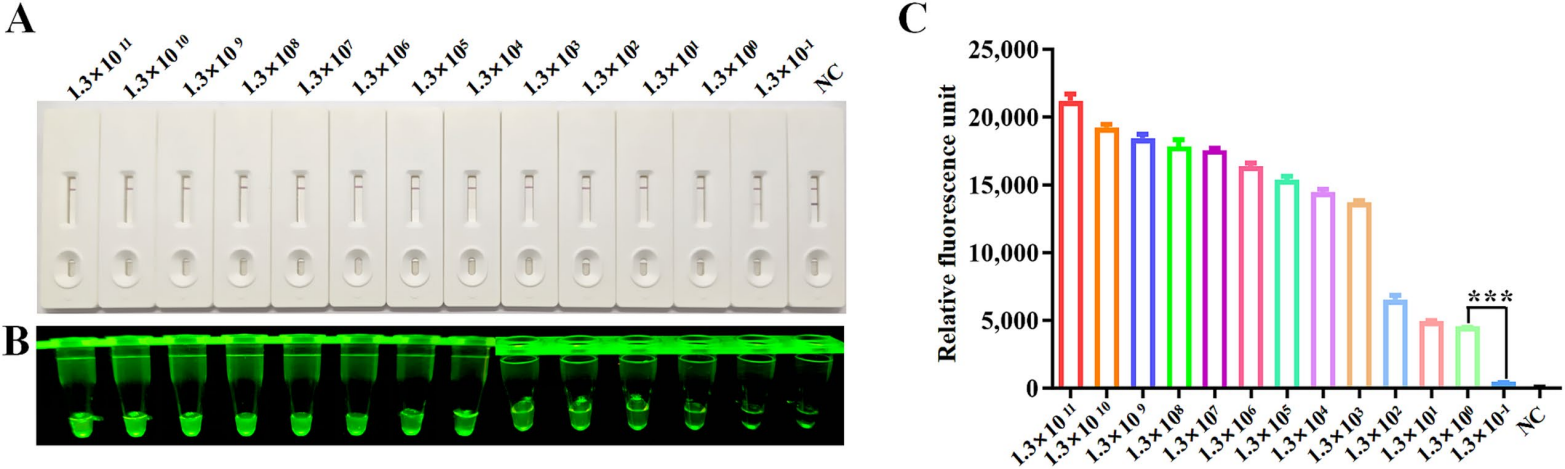
Sensitivity analysis. Sensitivity of CRISPR/Cas12a reaction for detecting the Rep gene with gradient concentrations from 1.3 × 10^11^ copies/μl to 1.3 × 10^−1^ copies/μl. NC indicates negative control. Sensitivity of RPA-CRISPR/Cas12a LFS detection **(A)** was assessed and verified by blue light detection **(B)** and fluorescence detection **(C)**.

The detection sensitivity of the CRISPR/Cas12a platform was evaluated using serially diluted plasmid standards (pcDNA3.1-N-MDPV VP3-Flag) ranging from 1.3 × 10^11^ to 1.3 × 10^−1^copies/μl. Following RPA amplification of each dilution series, CRISPR/Cas12a-LFS analysis revealed a detection threshold of 1.3 × 10^0^ copies/μl, evidenced by disappearance of the T-line band on lateral flow strips (Figure 4A) and marked differences in fluorescence intensity (Figure 4B) and relative fluorescence units (Figure 4C) between the 1.3 × 10^0^ and 1.3 × 10^−1^ copy groups.

### 3.5 Specificity of RPA-CRISPR/Cas12a detection method

**Figure 5:**
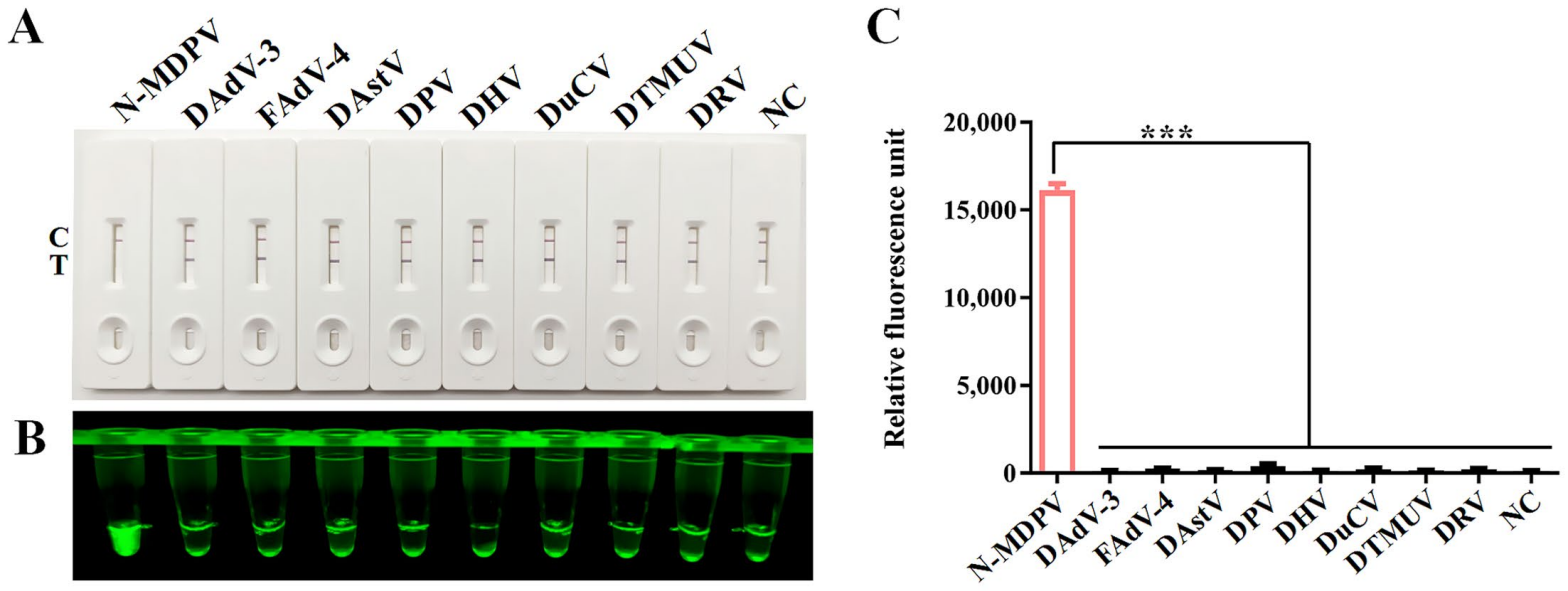
Specificity analysis. DNA of N-MDPV, DAdV-3, FAdV-4, DAstV, DPV, DHV, DuCV, DTMUV and DRV were used as templates for RPA-CRISPR/Cas12a reaction. The specificity of LFS detection **(A)** was assessed and verified by blue light detection **(B)** as well as fluorescence detection **(C)**.

The specificity of the CRISPR/Cas12a assay was verified by testing eight non-target duck viruses (DAdV-3, FAdV-4, DAstV, DPV, DHV, DuCV, DTMUV and DRV). As demonstrated through lateral flow strips (Figure 5A), blue light detection (Figure 5B), and fluorescence analysis (Figure 5C), N-MDPV DNA was exclusively detected, confirming method specificity with no cross-reactivity observed.

### 3.6 RPA-CRISPR/Cas12a-LFS detection of clinical samples

**Figure 6:**
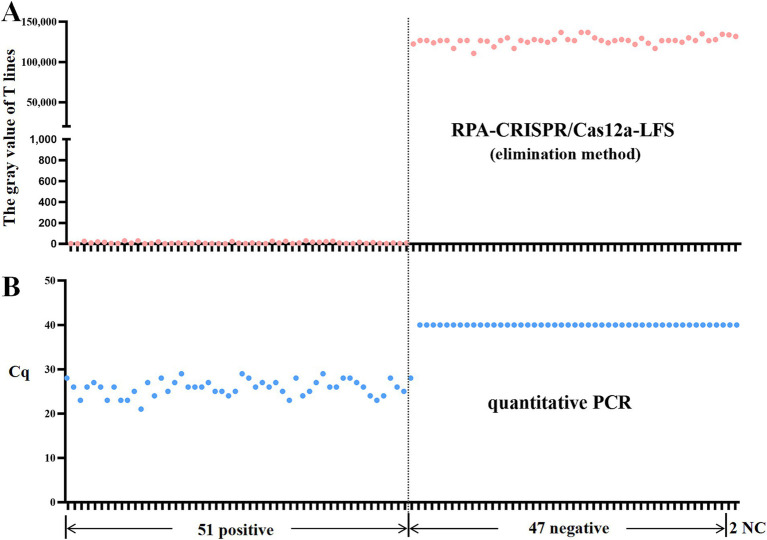
RPA-CRISPR/Cas12a-LFS detection of clinical samples. **(A)** Grayscale intensities of test lines (T) were quantified using ImageJ software, where detectable signals (presence of bands) indicated positive results. Comparative analysis of 98 clinical specimens by RPA-CRISPR/Cas12a-LFS **(A)** and qPCR **(B)** is displayed, with NC denoting negative controls.

The clinical utility of the RPA-CRISPR assay was validated using 98 duck samples. Following the detection principle outlined in Figure 1, lateral flow strip (LFS) results were quantified through grayscale analysis using ImageJ software (National Institutes of Health, Bethesda, MD, USA) of test line (T) intensities, where visible bands corresponded to positive signals. Specimens were categorized into 51 positives and 47 negatives based on grayscale thresholds (Figure 6). Comparative analysis with our previously established qPCR assay demonstrated 98.98% concordance (Table 2), confirming the method’s diagnostic reliability for N-MDPVdetection in clinical settings.

**Table 2:** The performance of RPA-CRISPR compared with qPCR.

Detection method		qPCR			CR
		Positive	Negative	Total	
RPA-CRISPR/Cas12a-LFS	Positive	51	0	51	98.98%
	Negative	1	46	47	
	Total	52	46	98	

## 4 Discussion

The effective containment of N-MDPV outbreaks hinges on early viral detection, particularly in scenarios where the strain demonstrates high mutability and lacks vaccine-targeted antigens. While PCR/qPCR methods remain the gold standard for N-MDPV identification (11, 18), their reliance on sophisticated thermocyclers and skilled personnel limits field applicability. LAMP assays, despite improved sensitivity (21), face challenges including intricate primer design, narrow thermal requirements, and susceptibility to false positives. These limitations underscore the urgent need for field-deployable diagnostic tools that balance accuracy with operational simplicity.

Recombinase polymerase amplification (RPA) has gained prominence as an isothermal amplification method, leveraging recombinase enzymes, DNA polymerases, and single-strand DNA-binding proteins. This technology demonstrates enhanced resistance to PCR inhibitors compared to conventional methods—specifically, it exhibits higher tolerance to impurities in samples, allowing simply lysed samples to be directly used as reaction templates-while accelerating result generation to under 20 min (22). However, RPA-based detection shows limited concordance rates with reference methods and compromised accuracy in low-template samples (25). CRISPR-Cas systems, renowned for sequence-specific recognition, have been repurposed for molecular diagnostics through fluorescence- or lateral flow strip (LFS)-based readouts (14, 15). In avian virology, CRISPR diagnostics have been successfully adapted for avian influenza virus (AIV), duck hepatitis A virus 3 (DHAV-3), and novel duck reovirus (NDRV) detection (23, 24). Notably, no peer-reviewed reports exist on CRISPR-Cas applications for N-MDPV identification, highlighting a critical gap in current diagnostic capabilities.

This study engineered a CRISPR/Cas12a system targeting conserved regions of the N-MDPV VP3 gene, where multiple protospacer adjacent motifs (PAMs) were characterized. crRNA designs targeting these PAM motifs enabled efficient Cas12a activation. Through systematic optimization of reaction parameters (primer selection, Cas12a:crRNA ratios), the platform achieved direct visual readouts via blue light excitation and lateral flow strips (LFS), demonstrating a detection limit of three viral copies. The workflow—spanning nucleic acid extraction to lateral flow strip (LFS) interpretation—achieves completion within 60 min while avoiding reliance on specialized instrumentation or technical expertise. Specificity evaluations against eight avian pathogens confirmed exclusive detection of N-MDPV, with no cross-reactivity observed. Clinical validation using 98 field samples demonstrated 98.98% concordance with gold-standard qPCR assays, substantiating diagnostic reliability.

## Data Availability

The original contributions presented in the study are included in the article/supplementary material, further inquiries can be directed to the corresponding authors.

## Footnotes

^1^ https://www.ezassay.com/primer

^2^ https://www.ezassay.com/rna
